# High-Precision Optical Fiber-Based Lickometer

**DOI:** 10.1523/ENEURO.0189-24.2024

**Published:** 2024-07-16

**Authors:** Artur Silva, Paulo Carriço, Ana B. Fernandes, Tatiana Saraiva, Albino J. Oliveira-Maia, Joaquim Alves da Silva

**Affiliations:** ^1^Champalimaud Research, Champalimaud Foundation, 1400-038 Lisbon, Portugal; ^2^NOVA Medical School, Faculdade de Ciências Médicas da Universidade Nova de Lisboa, 1169-056 Lisbon, Portugal; ^3^Department of Neurology, University Hospital of Würzburg, 97080 Würzburg, Germany; ^4^Champalimaud Clinical Centre, Champalimaud Foundation, 1400-038 Lisbon, Portugal

**Keywords:** consummatory behavior, lick microstructure, lickometer, open source, optical

## Abstract

Quantifying and analyzing licking behavior can offer valuable insights into fundamental neurobiological mechanisms controlling animal consummatory behaviors. Lickometers are typically based on electrical properties, a strategy that comes with limitations, including susceptibility to electrical interference and generation of electrical disturbances in electrophysiological measurements. While optical lickometers offer an alternative method to measure licks and quantify fluid intake in animals, they are prone to false readings and susceptibility to outside light sources. To overcome this problem, we propose a low-cost open-source lickometer that combines a restricted infrared beam defined by optical fibers, with a poke design that allows easy access to the tongue while limiting access of other body parts and external light sources. This device also includes features for detecting nose pokes and presenting visual cues during behavioral tasks. We provide validation experiments that demonstrate the optical lickometer's reliability, high-sensitivity and precision, and its application in a behavioral task, showcasing the potential of this tool to study lick microstructure in combination with other techniques, such as imaging of neural activity, in freely moving mice.

## Graphical abstract

**Figure:**
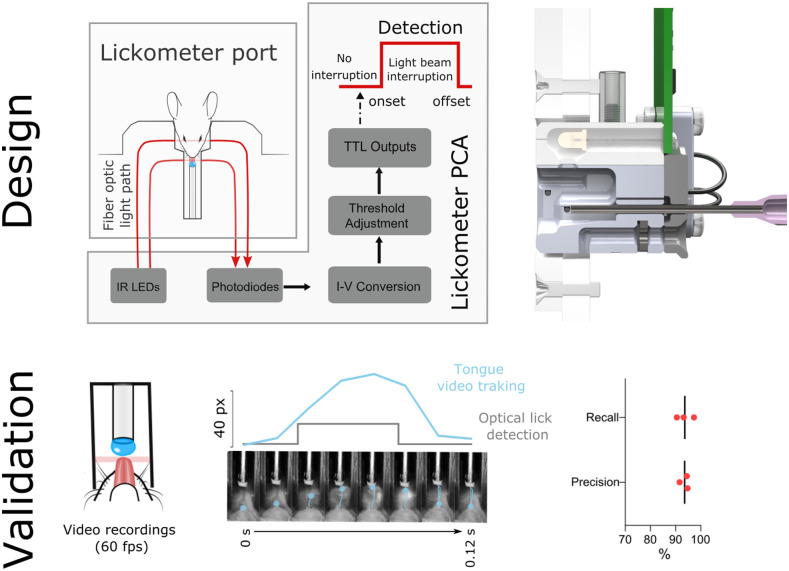
Visual Overview

## Significance Statement

Licking behavior is usually quantified using electricity-based lickometers. However, this type of lickometer is sensitive to electrical noise and may generate electrical artifacts that can be problematic in neurophysiological experiments. We present an innovative open-source optical lickometer that avoids these caveats while it provides a precise quantification of licking behavior. Its affordability, compatibility with existing behavioral setups, and ease of assembly make it accessible to researchers worldwide. By enabling high-precision data collection in freely moving mice, our device is a valuable tool to assess lick microstructure in behavioral tasks.

## Introduction

Consummatory behaviors are tied to basic survival mechanisms in animals. Behaviors like eating and drinking can reflect animals’ needs, motivation, and preferences. Adequate quantification and analysis of licks and its microarchitecture can provide important insight into fundamental neurobiological mechanisms that control feeding behavior (Araujo et al., 2008), and it can be a valuable tool in preclinical studies of related disorders such as obesity, anorexia, and addiction (Levin et al., 2010).

Early lickometers were developed >70 years ago, with a resistance-based design that relied on the completion of an electrical circuit when the tongue of the animal touches the spout to detect a licking event (Hill and Stellar, 1951; Williams and Teitelbaum, 1956). This design is still very popular and the basis of most commercially available and open-source lickometers (Raymond et al., 2018). A design which relies on the detection of capacitive changes when the tongue of the animal touches the liquid or spout was developed later (Wall et al., 1972) and has been also explored in open-source lickometers (Longley et al., 2017; Melo et al., 2022; Petersen et al., 2023).

To this day, the majority of lickometers used in research are variations of these designs. However, strategies based on electrical properties have several caveats, such as being sensitive to electrical noise and generating electrical artifacts in electrophysiological measurements hindering certain types of experiment (e.g., single unit neuronal recordings). Furthermore, lickometers that rely on the high resistance of the mouse are vulnerable to the development of pathways of lower resistance (by urine, feces, or water traces) that may lead to the detection of false licks (Schoenbaum et al., 2001). These limitations have motivated the appearance of other strategies to measure licks, such as the light-based strategies employed in optical lickometers. Optical lickometers are essentially based on the detection of licks by the interruption of an infrared light beam (Murakami, 1977; Schoenbaum et al., 2001), thus circumventing the caveats described for the electricity-based lickometers (Weijnen, 1998). Nevertheless, they present specific challenges. Beam breaks may happen while the animal is exploring the fluid without actual licks or because the beam is interrupted during fluid delivery. Also, light beam detection can be perturbed by external light sources, and commercially available optical lickometers tend to be more expensive than contact electricity-based lickometers.

However, recent open-source optical lickometers have been successfully implemented to quantify rodent drinking in a homecage environment (Frie and Khokhar, 2019; Godynyuk et al., 2019). Although more affordable, the design available was vulnerable to being interrupted by body parts other than the tongue, leading only to a moderate correlation between optical beam breaks and solution consumption (Godynyuk et al., 2019).

Here we describe a new affordable, open-source optical lickometer that provides individual lick quantification with high sensitivity and precision in freely moving mice, while avoiding the limitations of electricity-based lickometers. We used optical fibers to have a very small optical beam, and we positioned it close to the sipper that rests inside a slit designed to ensure that the light beam is interrupted by the tongue, and not other body parts, while simultaneously reducing external light interferences. Using a high-resolution camera, we were able to validate the high-precision and sensitivity of the system developed. Moreover, we characterized mice lick behavior in a task, focusing on the reliability of lick bout microstructure in freely moving mice.

Besides the ability to detect licks with high precision and sensitivity, our optical lickometer module also has a beam positioned to detect nose entries and has an LED positioned above the poke that can be used, for example, to present visual cues during behavioral tasks.

## Materials and Methods

### Optical fiber lickometer module

The proposed optical lickometer is composed of an electronic interface board, 3D printed parts housing the optical fibers, and a set of acrylic parts to support the lickometer. In addition to lick detection, this module also provides nose entry detection and control of an LED embedded in the device. A solenoid valve (HDI control, The Lee Company) can also be attached to the device and be externally controlled.

**Figure 1.**
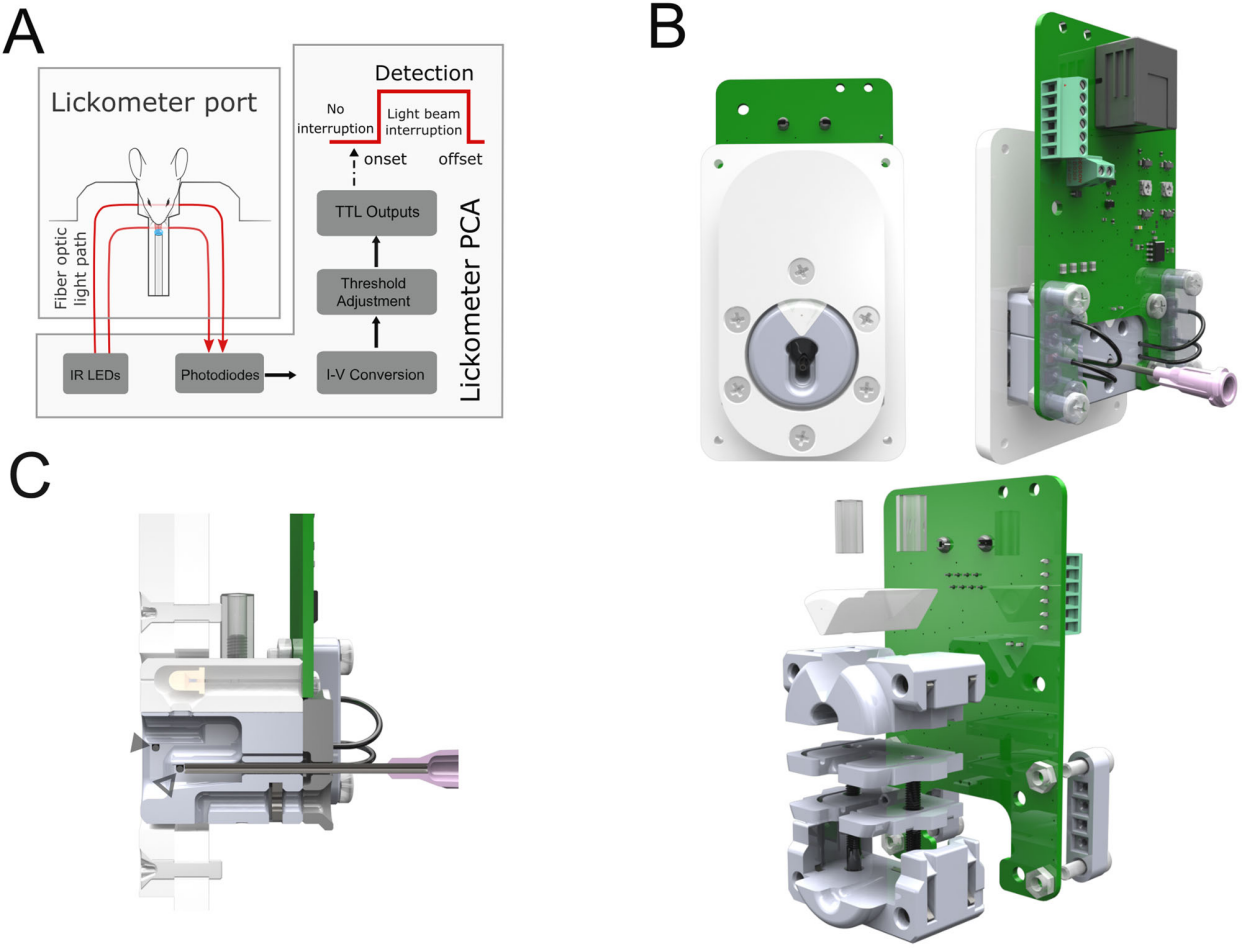
Optical lickometer module. ***A***, Schematics of the lickometer module highlighting the connection between the port and the lickometer printed circuit assembly (PCA). ***B***, Top left, Front view of the lickometer module. Top right, Back view of the lickometer module. Bottom, Exploded 3D view denoting the different parts of the lickometer module. ***C***, A sagittal cut with the details of the interior design of the lickometer port. Open and closed arrowheads denote the position of the lick detection fiber and head entry detection fiber, respectively. Extended Data Figure 1-1 is supporting Figure 1.

**Figure 2.**
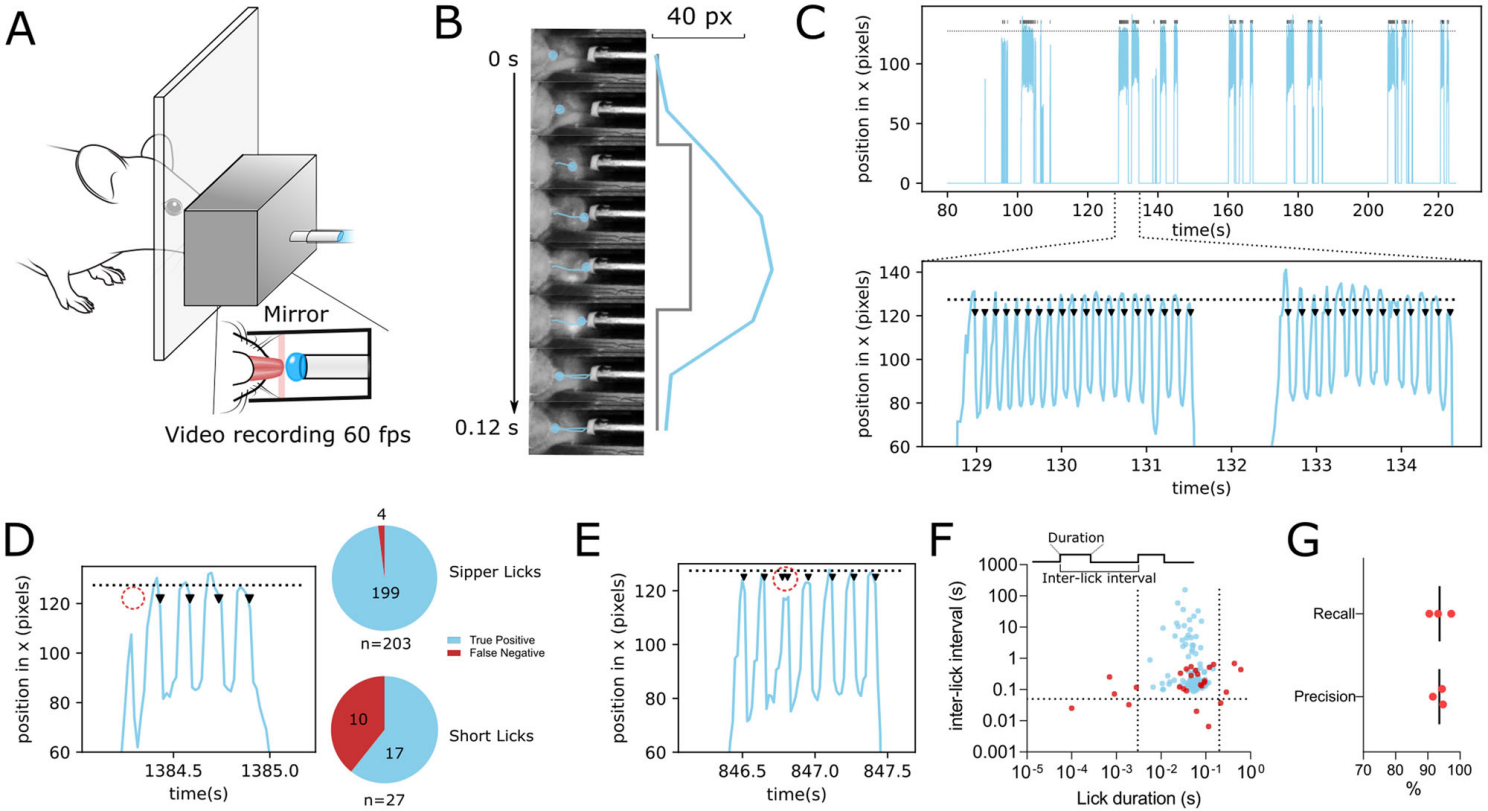
Optical lickometer has a high precision and recall. ***A***, Schematics of the setup to validate optical lickometer lick quantification using video. ***B***, Example of video frames during a lick with simultaneous quantification of the change in lickometer light path interruption (gray) and video-based tongue displacement (light blue). ***C***, Top, Example of the displacement of the tip of the tongue inside the lickometer (light blue) and the detection of licks by the lickometer (vertical black bars) during a session. Bottom, Detail of two bouts of licking (here lickometer licks are represented by arrowheads). Horizontal dashed line represents the position of the sipper. ***D***, Left, Example of a false-negative lick (red dashed circle). Right, Quantification of true-positive and false-negative licks depending on lick type. ***E***, Example of a false-positive lick (red dashed circle). ***F***, Scatterplot of ILI by lick duration for each lick. True-positive licks are depicted as light blue circles (*n* = 216) and false-positive licks as red circles (*n* = 26). Dashes lines represent the cutoffs used to exclude licks which have ILIs and lick durations that are outside of the true licks distribution. ***G***, Precision and recall of optical lickometer licks when compared with video-determined licks (*n* = 3).

The electronic interface board comprises a pair of two surface-mounted infrared LEDs, two surface-mounted photodiodes with their respective photodetection circuits, a 3 mm LED, a valve holder, and interface ports for external control signals and power supply (Figs. 1, 2). The lick and nose entry detection rely on interrupting an optical beam, where infrared LED light is emitted through an optical fiber, and a photodetector also coupled to an optical fiber receives and detects this beam. This design was based on the lickometer developed by Isett et al. (2018) for head-fixed experiments. The photodetector proportionally converts the received light into current, which is then converted to voltage using a transimpedance amplifier. A comparator with an adjustable threshold voltage controlled by a potentiometer is used to calibrate the optical detection trip point. Finally, digital buffers are used to generate the transistor–transistor logic (TTL) output signals (Fig. 1*A*). In this way, lick detection can be conveyed easily to any equipment capable of receiving TTLs, allowing online lick detection with high precision and sensitivity.

10.1523/ENEURO.0189-24.2024.f1-1 Figure 1-1 Layout of the lickometer PCB depicting the main circuitry blocks and interface connections. Download Figure 1-1, TIF file.

The design of the poke structure ensures that the lickometer beam can only be interrupted by the animal's tongue. This is achieved by placing the spout in a hollow structure, recessed in the poke structure, reached only through a tongue protrusion movement (Fig. 1*B*,*C*; Oliveira-Maia et al., 2008). The optical beam for nose poking is positioned near the poke entry (Fig. 1*C*), to easily detect nose entries.

Multiple 3D parts are required to assemble the poke device itself, but also to guide and position the optical fibers used (Fig. 1*B*). Two optical fiber segments are employed for each beam detector. For the first fiber segment, one end is positioned in close proximity with the LED case, in order to minimize power losses. The other end is aligned with the second optical fiber, which receives the light from the first beam and guides it as closely as possible to the photoreceiver (Fig. 1*C*). The process of placing and aligning the optical fibers is simplified due to the built-in grooves and holes in the 3D parts where the fibers are located. This enabled a good reproducibility and optimization of the fiber coupling, without resorting to more expensive solutions like preassembled fiber-coupled or fiber-pigtailed LEDs.

Two additional acrylic parts (Fig. 1*B*) are used to provide support and make it easier to attach the lickometer module to the wall of an operant box. The device is adaptable to fit onto any flat wall up to 5 mm thick. A flush fit is ensured by selecting the corresponding acrylic thicknesses of these parts, according to the operant box wall. To install, users will need to cut out an oval aperture and drill four holes at the designated position, securing the device to the wall using screws. An example of an operant box acrylic wall panel cutout is available for reference (Extended Data 1-1).

All the necessary electronic circuitry designs and production files, CAD files for 3D printing and laser cutting to assemble a complete lickometer, and poking device for freely moving mice in a behavior cage are available (Extended Data 1-1). These files are also freely available online at https://github.com/fchampalimaud/optical-lickometer.

10.1523/ENEURO.0189-24.2024.d1 Extended Data Design files and assembly instructions for the mechanical parts and printed circuit board of the proposed optical lickometer. Download Extended Data, ZIP file.

### Assembly instructions

To build the proposed device, the initial step consists of assembling the electronic components on the printed circuit board. This can be done manually either by using a soldering iron or by using a reflow oven, after applying solder paste and placing the components on the printed circuit boards. The next step involves the production of the designed 3D mechanical parts. The mechanical files provided (Extended Data 1-1) were designed for 3D printing using fused deposition modeling technology (selective laser sintering or stereolithography printers can also be used) and for laser cutting the acrylic parts (optionally CNC machining can also be employed).

A 500 µm diameter fiber with an optical grade plastic light guide was used (57-097, Edmund Optics) due to its bending, robustness, and ease of cutting when compared with their counterparts, glass fibers. The cutting process for the various fiber pieces was performed using a cutting block (54-013, Edmund Optics). The fibers are then guided along the 3D grooved sections of the fiber holder parts being positioned as close as possible to the light emitters and receivers. The poke 3D parts were assembled together and attached to the electronic board, along with the alignment parts, using screws secured to inner nuts integrated in the 3D parts. A 16 G blunt-end tip needle (847.356.0321, SAI Infusion Technologies) is used as a spout, and users have the option to trim the spout and attach directly to the water delivery tube or utilize it through a Luer lock fitting. Assembly instructions with example pictures are available (Extended Data 1-1).

Following the assembly, the alignment and coupling efficiency, due to the positioning of the fibers, can be validated by measuring the voltage at the output of the transimpedance amplifiers when the beam is not broken. The voltage level should be high enough to trigger the comparator and set the output to a high state. If this condition is not verified, the onboard potentiometers can be used to tune the optical beam detection threshold value, so that the light beam can be detected.

### Interface and control

For interfacing the lickometer device, users can choose between an Ethernet RJ connector and a screw terminal, with the respective pinouts depicted in Figures 1 and 2. These connections offer the flexibility for the user to select among a diverse range of hardware to interface this device that best suits the specific setup user requirements. The RJ connector pinout is compatible with other open-source behavioral hardware, namely, Bpod https://github.com/sanworks/Bpod-CAD (does not support the nose poke detection feature) and Harp Behavior https://github.com/harp-tech/device.behavior. The screw terminal interface also enables connection to other open-source development boards such as Arduino or Raspberry Pi.

### Animals

Male and female C57Bl/6 mice were used in the experiments described. All animal procedures were performed in accordance with the Champalimaud Foundation animal welfare and ethics review body and the Portuguese national food and veterinary agency. Mice were kept in light/dark cycle with the Zeitgeber Time 0 at 8:00 A.M. Experiments were performed during the light part of the cycle.

### Validation of lick detection by the optical fiber lickometer module

To validate the lickometer, we compared events detected by this module with licks detected by video acquired at a 60 Hz frame rate using a Flea3 USB3 monochromatic camera (Teledyne Flir). To be able to visualize the licks, we opened the bottom part of the lickometer module, just enough to see the opening of the poke and full extent of the channel that connects the poke to the sipper. We used a tilted mirror to transmit the bottom view of the lickometer module to the camera. We used a 16 G blunt needle (SAI Infusion Technologies) as a sipper. This blunted needle was introduced through the hole that exists in the back of the module to align the sipper with the light beam. The tip of the blunted needle rested ∼1.2 mm posterior to the center of the lickometer light path. The blunted needle was connected by a plastic tube to a glass syringe, controlled by a syringe pump.

Three mice were water deprived and trained to drink water through our lickometer module. We used two male mice and one female mouse. To ensure hydration, 0.8–1 ml of water was given to the mice in their homecage. Water volume was increased if necessary to keep mice weight >90% of the baseline weight.

Mice were first habituated to the boxes where the lickometers were. During this first day, 5 µl of water rewards were delivered with an intertrial interval (ITI) sampled from an exponential decay function with a mean of 18 s (limited to a minimum of 2 s and a maximum of 30 s). In the following sessions, rewards were delivered after a nose entry was detected by the lickometer module but respecting the same ITI distribution. Mice were trained for three sessions during which they learned to perform nose entries and licks to obtain water.

The behavioral task was implemented using an open-source multipurpose board (Harp behavior v2.0, Champalimaud Foundation) and Bonsai (Lopes et al., 2015), an open-source visual programming language based on ReactiveX programming to acquire the lickometer data obtained through the Harp behavior board and align it with video stream.

The data presented here was collected after these three sessions, on a single session for each mouse, from which we acquired video and lick detection data. We used DeepLabCut (Mathis et al., 2018; Nath et al., 2019) to detect the nose and tip of the tongue of each mouse. This allowed us to automatically detect video-based bouts of licking and compare the trajectory of the tongue with the detection by our optical lickometer.

To estimate the precision and sensitivity of the lickometer, we did a random sampling of 10 video-determined lick bouts per mouse. We determined the initiation and end of each lick by performing a frame-by-frame visual analysis, while being blind to the lickometer event detection data, and classified licks as “short” or “sipper” depending if they were too short or long enough to reach the sipper, respectively. Lickometer licks were classified as false negative if a lickometer event was not found within the interval determined by the initiation and finish of a lick. False-positive licks corresponded to lickometer events that occurred during a lick that had a previously recorded lickometer event or that occurred outside the time interval of a lick.

### Testing the use of the optical fiber lickometer module to quantify licking behavior in freely moving mice

#### Behavioral task

To test the viability of the optical lickometers to quantify licks in behavioral task, we trained 22 food restricted C57BL/6 mice (16 males and 6 females, weight 85% of baseline weight) to lick a sweetened solution [sucralose 0.04% (w/v)] provided through the optical lickometer sipper.

In this task, 5 µl of the sweetened solution was delivered when the first lick of a lick bout was detected by the optical lickometer. Rewards were made available with an ITI sampled from an exponential decay function with a mean of 18 s (limited to a minimum of 2 s and a maximum of 30 s).

The training schedule was composed of one or two sessions of habituation, where rewards were delivered without being triggered by the first lick. This allowed mice to learn that the lickometer port gave access to the sweetened solution and to start licking in the lickometer port. After the habituation sessions, mice were trained during a minimum of four sessions and a maximum of seven sessions with each session lasting 30 min. All mice tested acquired the behavior and learned to lick in the lickometer port to obtain the sweetened solution.

As in the previous experiment, the behavioral task was implemented using Harp behavior v2.0 (Champalimaud Foundation) and Bonsai (Lopes et al., 2015) to acquire the lickometer data and align it with video stream and fiber photometry data (see below). Bonsai files are freely available online at https://github.com/fchampalimaud/optical-lickometer.

#### Dopamine sensor imaging

To confirm that the optical lickometer module can be successfully integrated with in vivo measurements of brain physiology in freely moving mice, we expressed a dopamine sensor (dLight1.2) in the ventral striatum and recorded its activity using fiber photometry.

Surgeries were performed using a stereotaxic system (Kopf) to inject 500 nl of an AAV5.hSyn.dLight1.2 (Addgene) virus solution in the ventral striatum (+1.3 mm anteroposterior, ±1.25 mm lateral, and 3.93 mm deep from the brain surface). An optical fiber with a core diameter of 200 µm and a numerical aperture of 0.37 (Neurophotometrics) was implanted 0.1 mm above the coordinates of the injections. The fibers were fixed in place using cyanoacrylate and black dental cement (Ortho-Jet). One 0.0625 inch stainless-steel screw (Antrin Miniature Specialties) was attached to the skull to provide a scaffold to build a dental-cement–based cap that fixed the fibers to the skull.

#### Acquisition and analysis of dLight1.2 data

Fiber photometry was performed using a Neurophotometrics F3002 system, with an acquisition rate of 50 Hz for each channel (470 nm channel for dLight1.2 and 415 nm channel for isosbestic data). Raw traces of 470 nm and isosbestic data were corrected for bleaching by subtracting a lowess regression of each signal from the raw data (lowess function from the statsmodel python library, frac = 0.1, it = 3, delta = 20). Then a delta fluorescence was calculated by subtracting the isosbestic signal from the dLight1.2 signal. Finally, a *z*-score of this final trace was calculated.

### Data analysis

Analysis of experimental data, data plotting, and statistical analysis were performed in Python (3.7) and Prism (version 8).

Fisher’s exact test was used to compare proportions between groups, and Pearson’s correlation coefficient was used to quantify the linear association of two variables. One-way repeated-measures ANOVA was used to compare the means of repeated observations of a dependent variable. Two-way repeated-measures ANOVA was used to compare means of repeated observations between groups that have been split by two factors. The significance level was set at an *α* value of 0.05.

Data from validation experiments will be provided upon reasonable request.

## Results

### Validation of optical fiber-based lickometer lick detection using video tracking

To validate the ability of the developed device to detect licks, we performed an experiment where lickometer measurements were compared with video detection of tongue protrusions (Fig. 2*a*,*b*).

We found that lickometer events only happened during video-determined lick bouts and that there was a high consistency between video-determined licks and lickometer events (Fig. 2*c*). To estimate the precision and sensitivity of the lickometer, we did a random sampling of 10 video-determined lick bouts per mouse, which resulted in the analysis of a total of 230 licks (77 ± 4.5 licks per mouse). We found a mean of 72 ± 3.27 true-positive, 4.7 ± 2.1 false-negative (example in Fig. 2*d* and Movie 1), and 8.6 ± 3.8 false-positive licks (example in Fig. 2*e* and Movie 2), resulting in a precision of 89.5 ± 3.61% and a recall of 94.0 ± 2.42%. The proportion of false-negative licks was significantly enriched in “short” licks (licks that did not reach the sipper, 10/27 licks), while false-negative licks were a rarity within “sipper” licks (licks that reached the sipper, 4/203 licks; *p *< 0.0001; Fisher's exact test; Fig. 2*d*).

Regarding false-positive licks, we reasoned that some of these events could have characteristics that distinguish them from true-positive licks post hoc. For example, in the case of a double detection during a single lick, the interval between the two events might be obviously different from the normal interlick interval (ILI). Furthermore, in the case that mice break the light beam with their snout while exploring, the duration of the lick event may also be different from the usual duration of a lick. To explore this hypothesis, we plotted the ILI against the duration of each lickometer event for false- and true-positive lickometer events. We verified that indeed using inclusion criteria of lick durations between 0.003 and 0.2 s and/or an ILI >0.05 s reduced the number of false-positive licks to 5 ± 1.4, without changing the percentage of false-negative licks (Fig. 2*f*). Using this post hoc correction, we increased the precision to 94 ± 1.8% while maintaining the same recall value (Fig. 2*g*).

### Viability of the fiber-optic lickometer to quantify licks in freely moving mice

**Figure 3.**
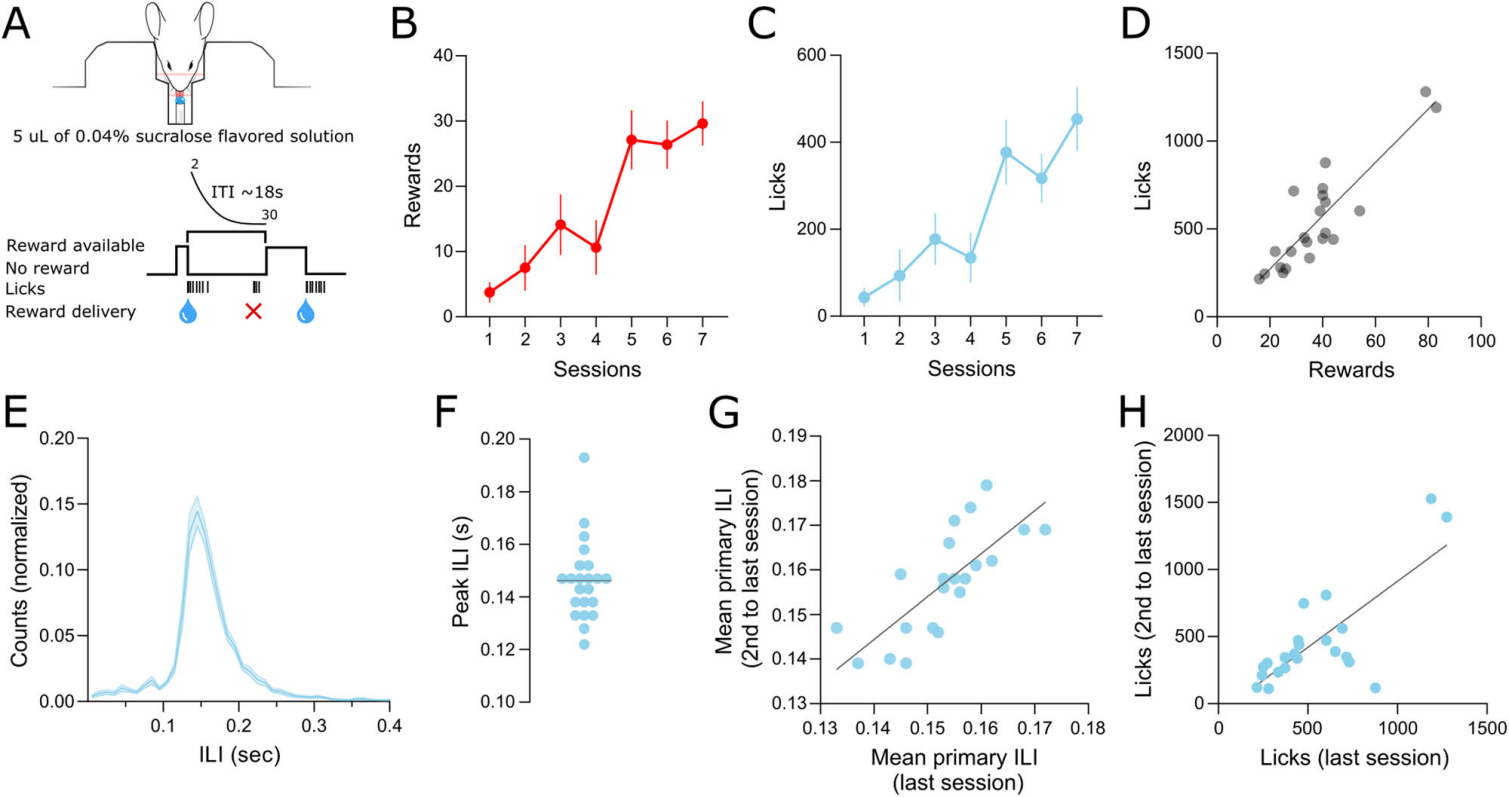
Lick quantification and microstructure is consistent across behavior training sessions. ***A***, Scheme of the behavioral task. ***B***, Number of rewards across training sessions (*n* = 8) and ***C***, number of licks across training sessions (*n* = 8) for mice that underwent seven training sessions. ***D***, Scatterplot depicting the correlation between licks and rewards for all mice (*n* = 22). ***E***, Distribution of the ILIs <0.5 s. ***F***, Peak ILI for each mouse (max value of the distribution of each mouse, *n* = 22). ***G***, Scatterplot depicting the correlation of the ILI between the two last training sessions (*n* = 22). ***H***, Scatterplot depicting the correlation of the total number of licks between the two last training sessions (*n* = 22). Error bars and shaded area denote SEM.

To test the viability of the optical lickometers to consistently quantify licks in the context of a freely moving behavioral task, we trained 22 food-restricted C57BL/6 mice (16 males and 6 females) to lick a sweetened solution provided through the optical lickometer sipper (Fig. 3*A*). Additionally, to confirm that the optical lickometer module can be successfully integrated with in vivo measurements of neuronal physiology, we expressed the dopamine sensor dLight1.2 (Patriarchi et al. 2018) in the ventral striatum of these mice and imaged its activity using fiber photometry.

Mice were trained from four to seven sessions (5.68 ± 1.26 sessions). In the subgroup of mice that were trained for a longer period (seven sessions, *n* = 8), there was a progressive increase in the number of rewards obtained (Fig. 3*B*; repeated-measures one-way ANOVA; *F *= 15.03; *p *= 0.0001; linear trend test: *p *< 0.0001) and a corresponding increase in the number of licks per session (Fig. 3*C*; repeated-measures one-way ANOVA; *F *= 9.39; *p *= 0.0018; linear trend test: *p *< 0.0001). Furthermore, in the last training session of all mice, the number of licks was highly correlated with the number of rewards (Pearson’s *r *= 0.88; *p *< 0.0001; *n* = 22; Fig. 3*D*).

To evaluate the ability of the fiber-optic lickometer to adequately capture lick microstructure, we analyzed the ILIs within each lick bout focusing on ILIs <0.5 s. We found a tight distribution with most ILI falling on the 0.1–0.2 s interval as previously reported (Boughter et al., 2007; Raymond et al., 2018; Fig. 3*E*). The mean modal ILI was 0.146 ± 0.015 s (Fig. 3*F*).

The mean primary ILI (mean ILI in the 0.05–0.25 s interval) was consistent within mice, with a high correlation between sessions (Fig. 3*G*; Pearson’s *r *= 0.78; *p *< 0.0001) when comparing the values of the last and second to last training sessions. Also, the number of licks detected in the two last sessions was consistent (Fig. 3*H*; Pearson’s *r *= 0.77; *p *< 0.0001).

**Figure 4.**
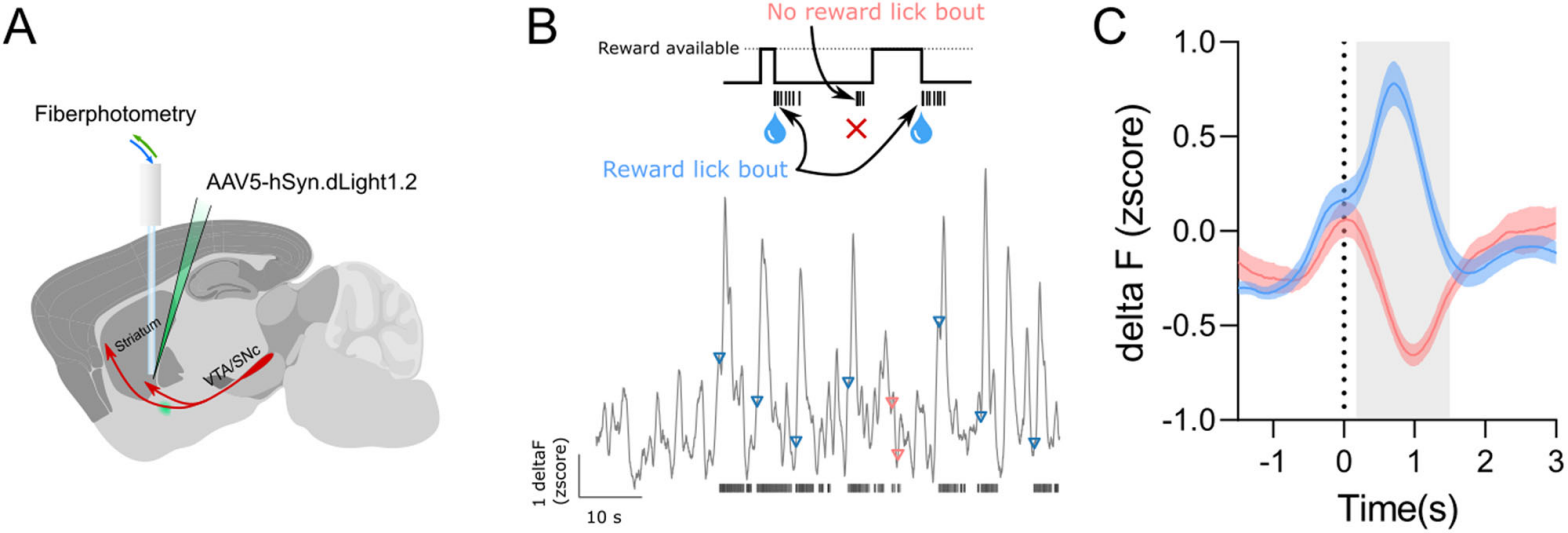
Use of the optical lickometer to align licking behavior with dopamine sensor imaging. ***A***, Schematics depicting dopamine imaging in the ventral striatum using dLight1.2 and fiber photometry. ***B***, Example of a dLight1.2 trace and its relation with the lick behavior. Blue arrowheads and red arrowheads denote where the dLight1.2 trace aligns with the first lick of a bout that led to a reward, and the first lick of a bout that did not lead to a reward, respectively. Vertical black lines represent licks detected by the lickometer. ***C***, Perievent time histogram of dLight1.2 data aligned to the first lick that led to a reward (blue trace) and the first lick that did not lead to a reward (red trace, *n* = 22). Gray shaded area corresponds to the time points where post hoc tests revealed that the mean of rewarded trials was significantly different from the mean of unrewarded trials. Blue and red shaded areas denote s.e.m.

By measuring dopamine release in the ventral striatum while mice licked, we were able to show that the lickometer can be adequately integrated with methods to measure neuronal function in freely moving mice (Fig. 4*A*). Given the design of our task, if mice started a lick bout, they would only gain access to a sweet solution reward when the ITI had passed (Fig. 4*B*, top). Using licks detected by the lickometer, we found significant differences in dopamine levels in the ventral striatum when comparing rewarded and nonrewarded licks (two-way repeated-measures ANOVA; time: *F*_(225,4725)_ = 7.082, *p *< 0.0001; type of trial: *F*_(1,21)_ = 15.73, *p *< 0.0007; interaction: *F*_(225,4725)_ = 26.22, *p *< 0.0001). We observed the expected increase in dopamine levels provoked by the licking of a rewarding solution, and a significant reduction of dopamine levels when mice start a licking bout but no reward was available (Fig. 4*C*; Schultz, 2016; Patriarchi et al., 2018).

**Movie 1.** Movie clip depicting a false-negative short lick. Movie displayed 12× slower than real time. [View online]

**Movie 2.** Movie clip depicting a false-positive lick. Movie displayed 12× slower than real time. [View online]

## Discussion

In this study, we developed and validated a novel open-source optical lickometer designed to accurately quantify licking behavior in freely moving mice. By utilizing fiber-optic infrared light beams and carefully designed structural features, we obtained a high level of consistency between lickometer-detected events and video-confirmed licks, with a low percentage of false positives and false negatives, resulting in a precision and recall higher than other open-source optical-based methods (Godynyuk et al., 2019) and strain gauge-based force lickometers (Crompe et al., 2023).

The poke design we presented could raise some concerns regarding its use in experiments involving mice with head caps for recordings, imaging, or optogenetic manipulations. However, in our validation experiments, we successfully used mice with bilateral optical fiber implantation, tethered to patch cords, without compromising lick detection. Licks were adequately detected with lick number increasing during learning and a high correlation between the number of licks and the number of rewards obtained.

Furthermore, lick detection was consistent within mice, and the ILI distribution had a shape similar to what has been reported by other researchers (Boughter et al., 2007; Raymond et al., 2018). Importantly, we did not find the “harmonics” seen by others (Boughter et al., 2007; Raymond et al., 2018) at values multiple of the mean modal ILI. These “harmonics” result from not detecting some licks within a bout, and their absence is in accordance with the very low percentage of false-negative licks we found in the previous experiment. However, the mean modal ILI was longer than previously reported (Boughter et al., 2007; St. John et al., 2017; Raymond et al., 2018). Although the view of licking as a very invariable motor output from central pattern generators persists, the stability of the ILI is also related to aspects that are kept fixed across experiments (Weijnen, 1998). For example, it has been shown that ILI increases as the distance between the animal and the drinking tube increases (Corbit and Luschei, 1969; Davis and Smith, 1992). We propose that this was the case with our lickometer: The design of the poke with a slit and a retracted sipper, while allowing for higher precision, increased the distance between the mouse and the sipper in comparison with other lickometers, resulting in an increased ILI.

This characteristic, along with other limitations of the different lickometers, is often relative. They depend on the different experimental objectives and may naturally emerge from experimental trade-offs, as was the case here. Nevertheless, due to the open-source nature of this device and the consequent availability of the design files, it is possible to adjust the device taking into account the specific objectives of each experiment.

While our device may seem more complex in comparison with other open-source lickometers, it can be easily assembled even by users without technical skills. The assembly process does not require special hardware and is guided by detailed assembly instructions. Furthermore, its embedded fiber alignment design combined with the adjustable optical detection trip point makes it straightforward to get a consistent aligned light beam. Also, the design with a screw terminal interface and a connector compatible with open-source behavioral hardware facilitates the incorporation of the lickometer into already existing behavioral setups and the combination with other data sources as we demonstrated with our fiber photometry results.

In addition to this, its compact design enables the assembly of multiple lickometer devices in a single behavioral box. Also, if a multichannel sipper is used, more than one solution type can be offered in the same module.

In summary, here we present a novel open-source, affordable, high-precision optical lickometer which can provide detailed data on licking microstructure of freely moving mice. By combining precise lick measurements with dopamine sensor imaging, we have illustrated the utility of our device, which can assume an important role in a variety of behavioral experiments where licking is a relevant output.

## Synthesis

Reviewing Editor: Mark Laubach, American University Decisions are customarily a result of the Reviewing Editor and the peer reviewers coming together and discussing their recommendations until a consensus is reached. When revisions are invited, a fact-based synthesis statement explaining their decision and outlining what is needed to prepare a revision will be listed below. The following reviewer(s) agreed to reveal their identity: Marie Doyle, Jude Frie.

Your manuscript was reviewed by two experts. They suggested some revisions. Their reviews are given below. Please revise your manuscript to address all concerns that were raised. Thank you for sending your work to eNeuro!

Also, please provide a link to your Bonsai repository so the files can be checked at the time when you resubmit your manuscript. You can send the link to me at mark.laubach@gmail.com.

Reviewer 1

This manuscript describes a new open-source IR beam-based lickometer system for measuring mouse drinking behaviors in freely moving animals. A significant advantage over existing IR systems, both commercially available and open-sourced, is the ability for the design to detect head entries vs actual licks. Overall the manuscript is well written and provide detailed build instructions for the tool. There are several minor points to address:

General:

Adding details for how the lickometer is housed in a behavioral box would be helpful for researchers in understanding how this tool can be applied to their behavior. For example, this tool seems ideal for use in operant boxes. Does it fit in an operant box wall panel? Are specialized behavioral boxes required to be built?

Methods:

- Minor: List the sex and species of animals used for the experiments in the "Animals" section. It is listed throughout but would be clearer to add "male and female C57Bl/6" here as well.

- Minor: List statistical tests used in the Data Analysis section.

Results:

- Minor: Add the percent of mice that acquired training (or number that did not acquire). Ie, does the design of the lickometer port result in/contribute to poor acquisition of behavior?

- Minor: Add a statistical comparison for dLight signal in Figure 4C rather than just stating the signal went "up" or "down".

Reviewer 2

The present manuscript describes the construction and validation of an optical fibre-based lickometer that effectively solves the previously identified issues of non-lick obstructions associated with other optical lickometers. The provided build instructions are comprehensive, making it possible for researchers to construct the design with relative ease. Additionally, the included PCB and .STLs follow proper design practices, ensuring successful fabrication, and enable robust integration with other equipment, as illustrated by the successful combination with fibre photometry described. I can confidently recommend the publication of this manuscript following very minor edits and the availability of the final repository mentioned in the methods section.

Edits:

1. Please include the original design files for the PCB such that it may be customized.

2. Grammatical errors:

a. Sentence two of the significance statement contains a subject verb disagreement (i.e. "...this type of lickometers are...")

b. Paragraph four of the intro is missing a period at the end.

c. Sentence two of data analysis states "A p value >0.05 was considered statistically significant." Should be p<0.05.
