# Supplementary figures and images for: High-Precision Optical Fiber-Based Lickometer
Source: eNeuro. 2024 Jul 16;11(7):ENEURO.0189-24.2024. doi: 10.1523/ENEURO.0189-24.2024 (PMC11258538; doi:10.1523/ENEURO.0189-24.2024)

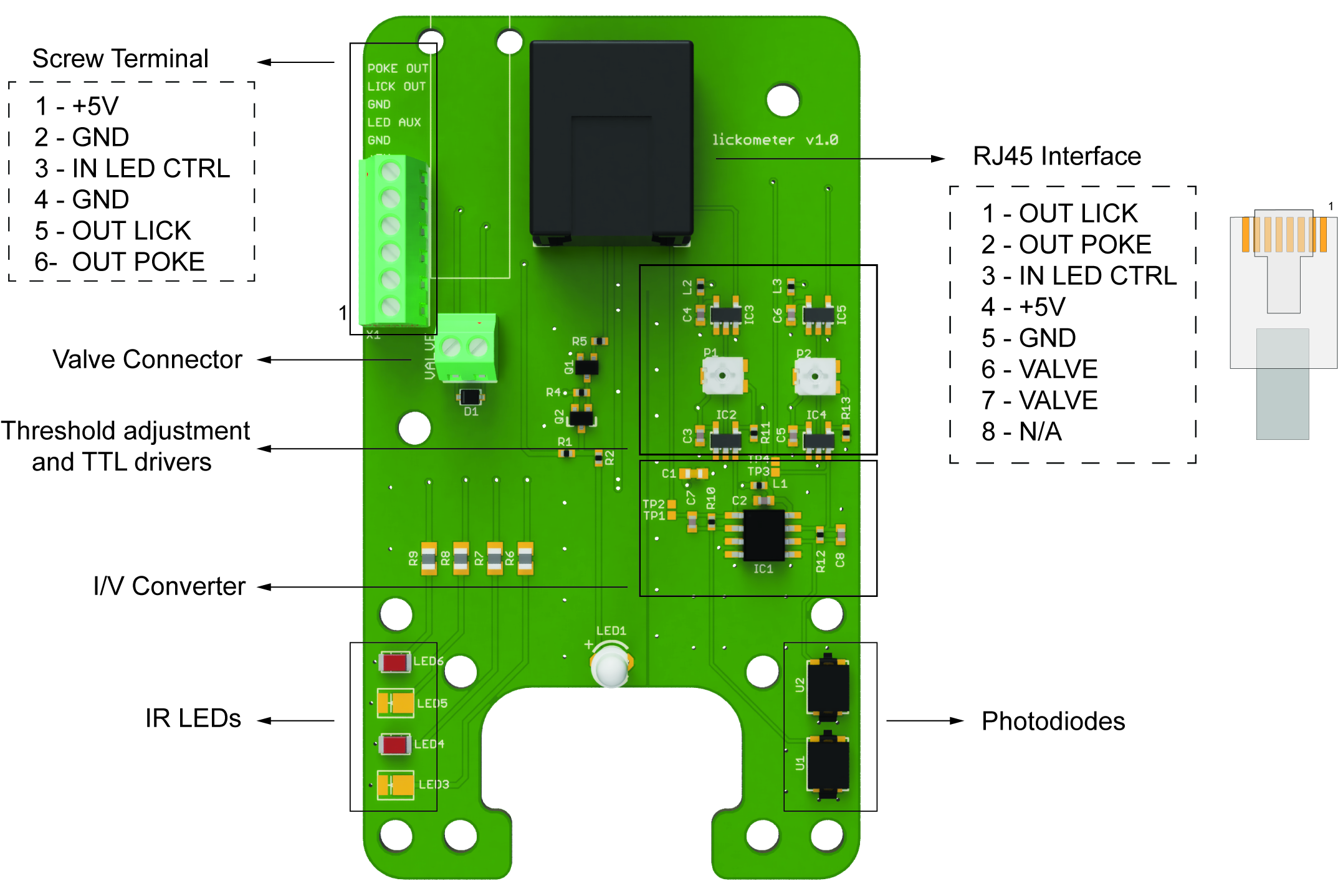

Supplement: Figure 1-1 — Layout of the lickometer PCB depicting the main circuitry blocks and interface connections. Download Figure 1-1, TIF file. [file eneuro-11-ENEURO.0189-24.2024-s006.tif]

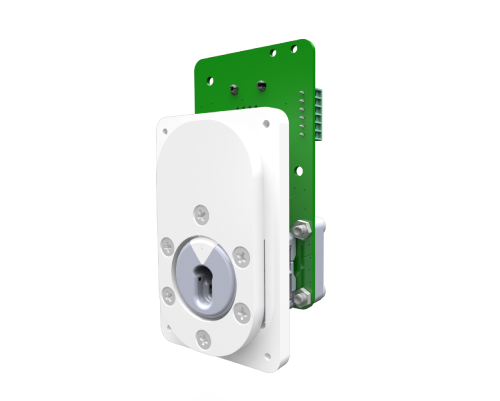

Supplement: Extended Data — Design files and assembly instructions for the mechanical parts and printed circuit board of the proposed optical lickometer. Download Extended Data, ZIP file. [file eneuro-11-ENEURO.0189-24.2024-s004.zip › Assets/lickometer.png]

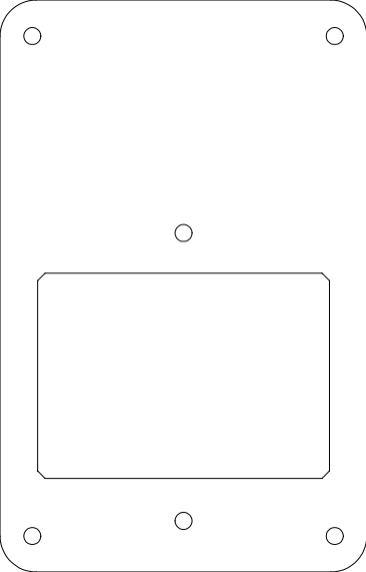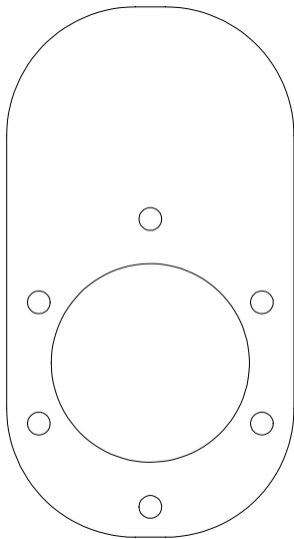

Supplement: Extended Data — Design files and assembly instructions for the mechanical parts and printed circuit board of the proposed optical lickometer. Download Extended Data, ZIP file. [file eneuro-11-ENEURO.0189-24.2024-s004.zip › Hardware/CAD/Acrylic_Plates_Lickometer_5mm_v1.pdf]

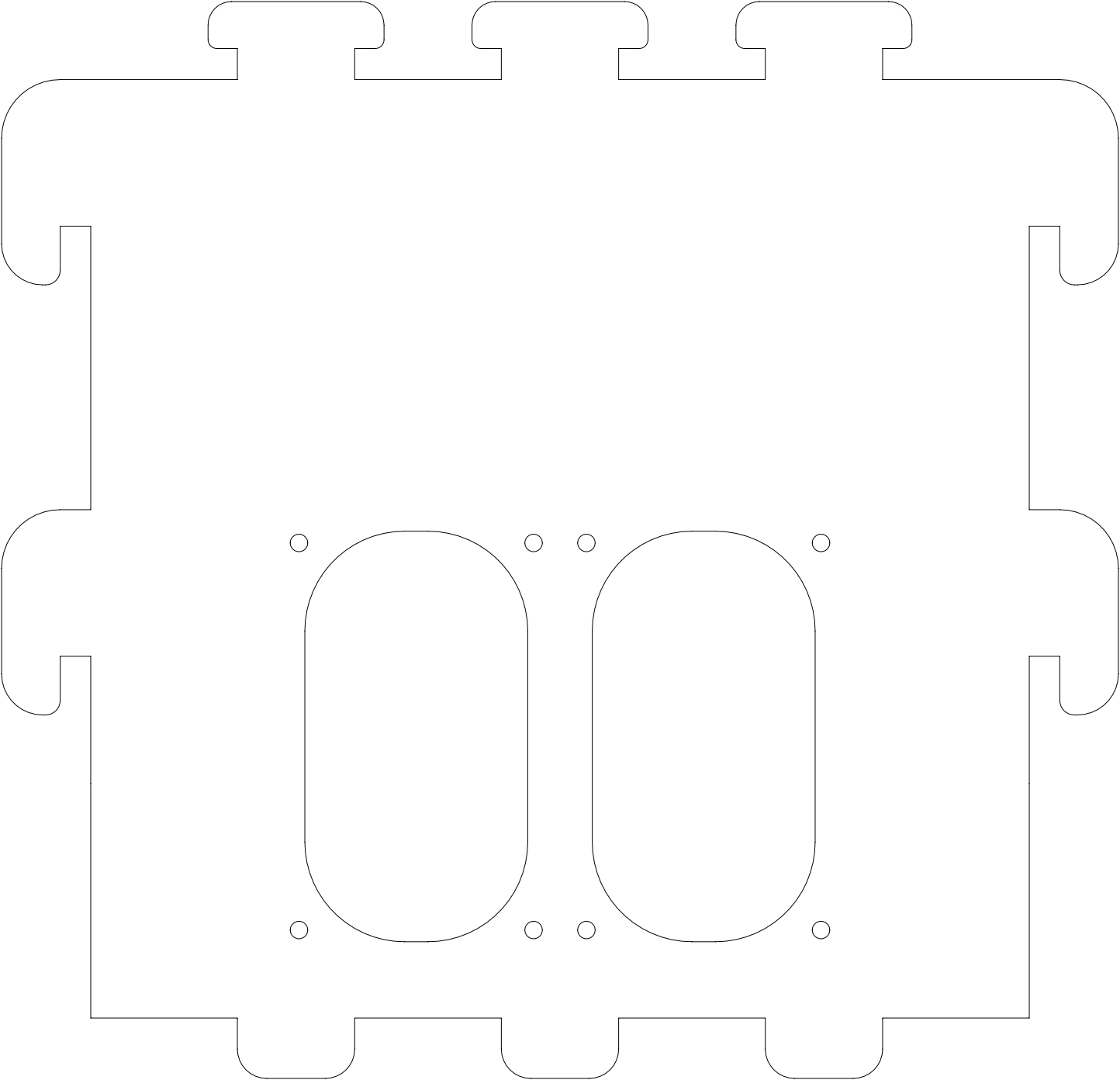

Supplement: Extended Data — Design files and assembly instructions for the mechanical parts and printed circuit board of the proposed optical lickometer. Download Extended Data, ZIP file. [file eneuro-11-ENEURO.0189-24.2024-s004.zip › Hardware/CAD/Acrylic_Plates_Panel_Mount_Example_5mm_v1.pdf]

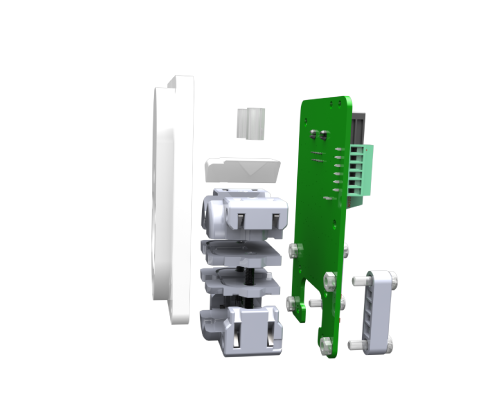

Supplement: Extended Data — Design files and assembly instructions for the mechanical parts and printed circuit board of the proposed optical lickometer. Download Extended Data, ZIP file. [file eneuro-11-ENEURO.0189-24.2024-s004.zip › Hardware/CAD/Lickometer_render_view.png]

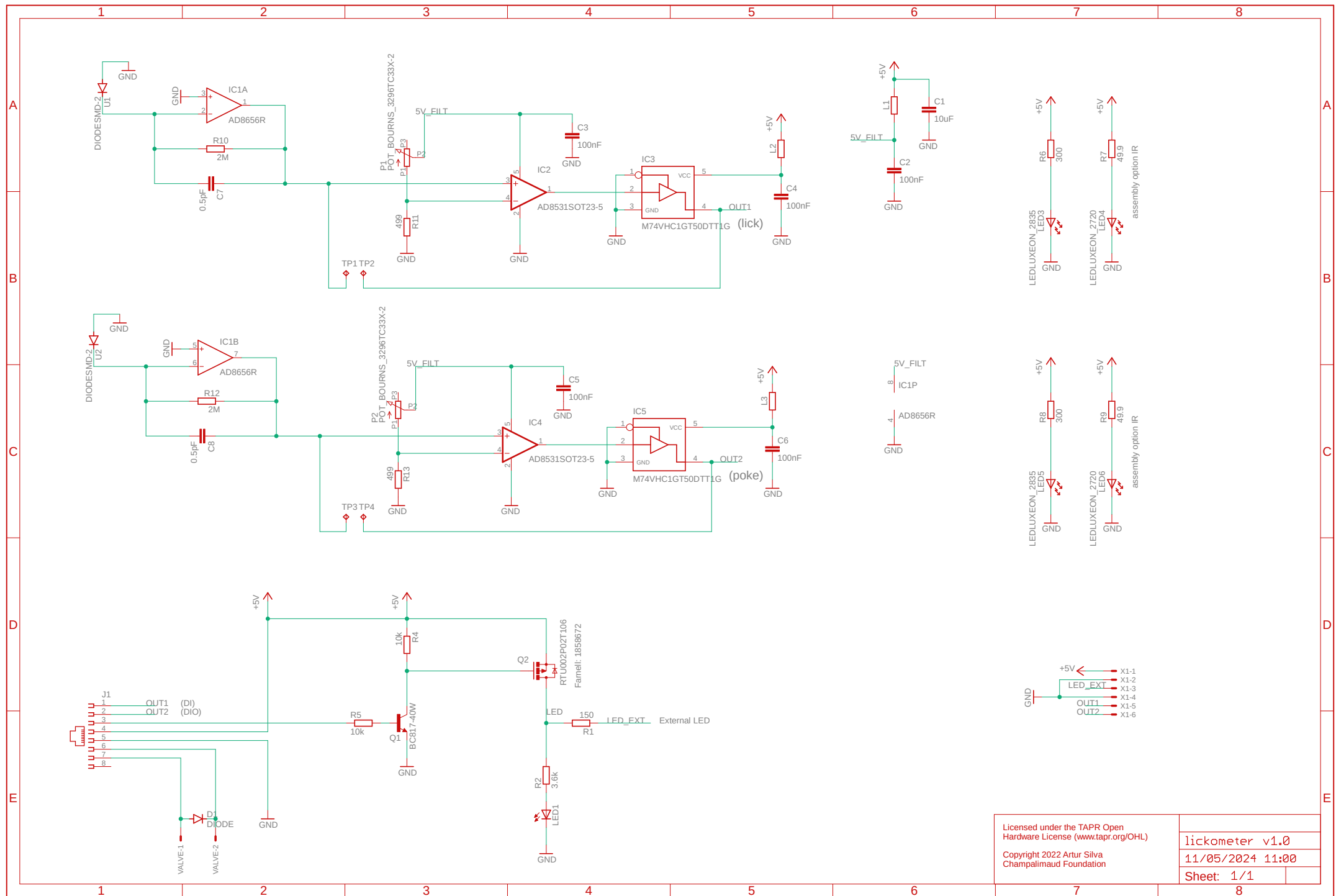

Supplement: Extended Data — Design files and assembly instructions for the mechanical parts and printed circuit board of the proposed optical lickometer. Download Extended Data, ZIP file. [file eneuro-11-ENEURO.0189-24.2024-s004.zip › Hardware/PCB/lickometer v1.0 - sch.pdf]
