# Supplementary material for: High-Precision Optical Fiber-Based Lickometer
Source: eNeuro. 2024 Jul 16;11(7):ENEURO.0189-24.2024. doi: 10.1523/ENEURO.0189-24.2024 (PMC11258538; doi:10.1523/ENEURO.0189-24.2024)

Lickometer Assembly instructions

1. Assemble the electronic components on the printed circuit board. This can be done manually either by using a soldering iron or by using a reflow oven, after applying solder paste and placing the components on the printed circuit boards. The bill of materials of the electronic board is available in the repository.

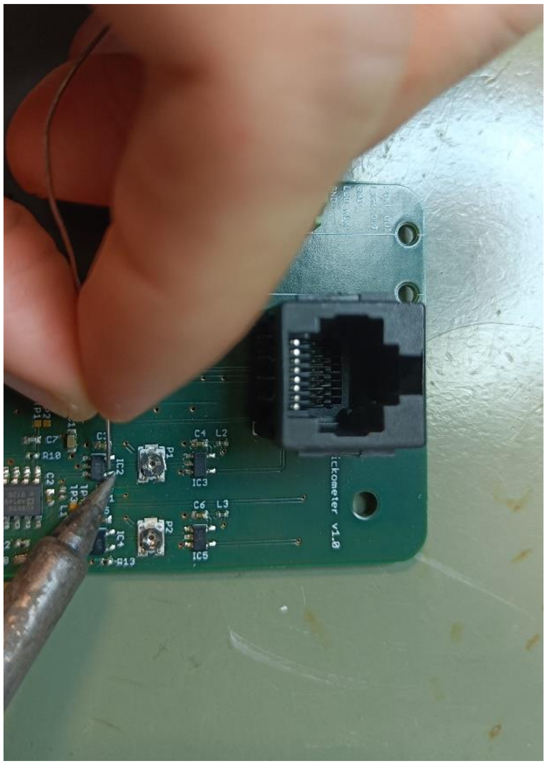

2. Laser cut the parts available in the repository. Use a 5 mm width acrylic sheet for the Acrylic_Plates_Lickometer_5mm_v1.pdf file. Perform M3 threading on the back acrylic part using a tap tool and on the front acrylic use a chamfer cut deburring drill to create countersunk holes. Use 2x M3 12 mm length countersunk screws to attach the acrylic parts.

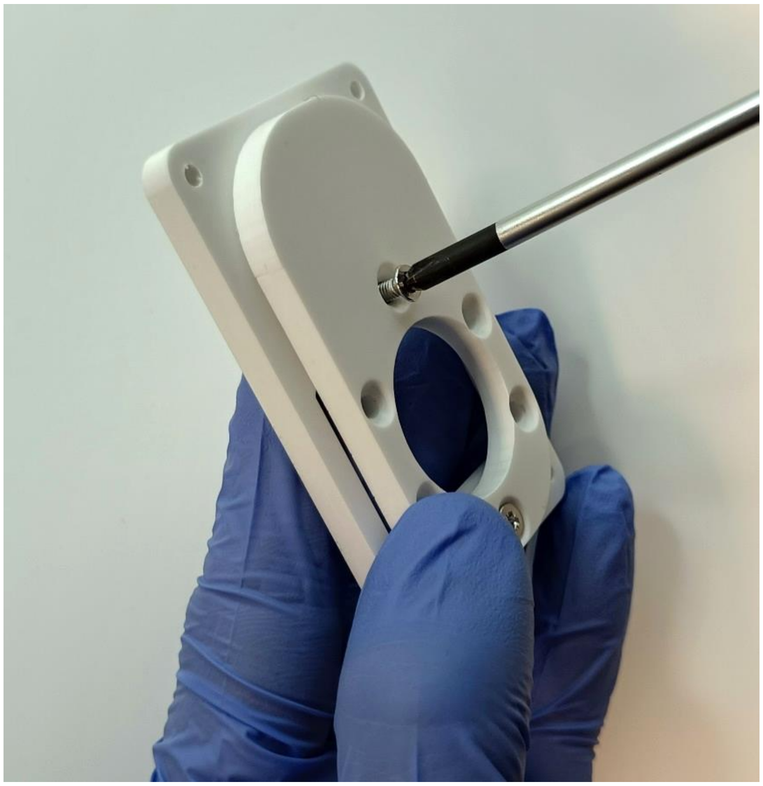

3. 3D print the STL files available in the repository, namely:

- (1) Lickometer_Dual_Detection_Optical_Fiber-1.STL
- (2) Lickometer_Dual_Detection_Optical_Fiber-2.STL
- (3) Lickometer_Dual_Detection_Optical_Fiber-3.STL
- (4) Lickometer_Dual_Detection_Optical_Fiber-4_F.STL - for female mice
- (4) Lickometer_Dual_Detection_Optical_Fiber-4_M.STL - for male mice
- (5) Lickometer_Dual_Detection_Optical_Fiber-5.STL
- (6) Lickometer_Dual_Detection_Optical_Fiber_Photo_Holder.STL
- (7) Lickometer_Dual_Detection_Optical_Fiber_LED_Holder.STL
- (8) Lickometer_Dual_Detection_Optical_Fiber-6.STL (optional)

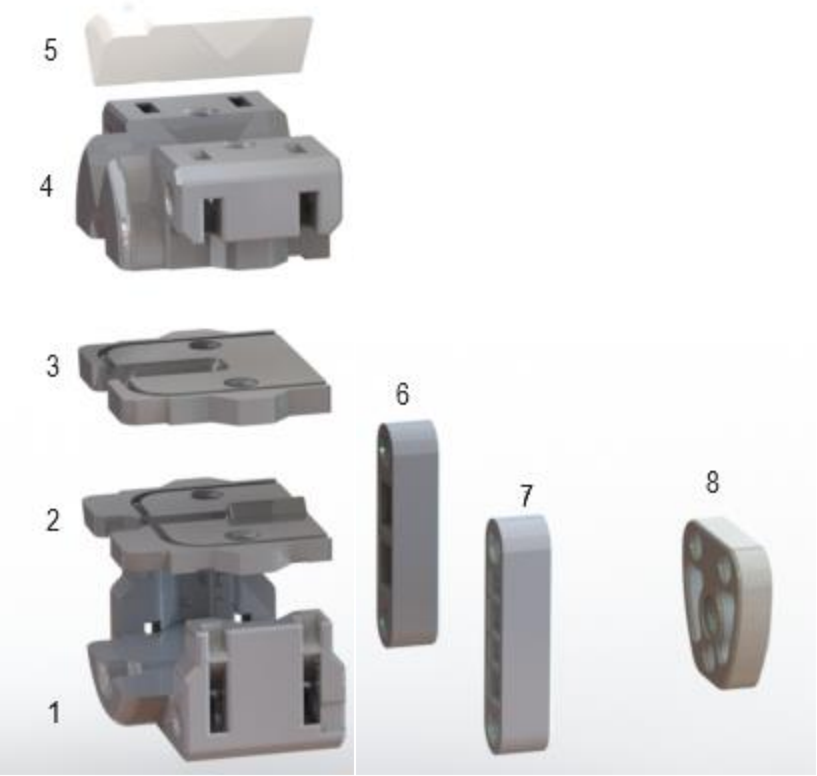

4. Cut four pieces of approximately 77 mm length of the 500 µm fiber diameter (57-097, Edmund Optics). The cutting process for the various fiber pieces is performed using a cutting block (54-013, Edmund Optics).

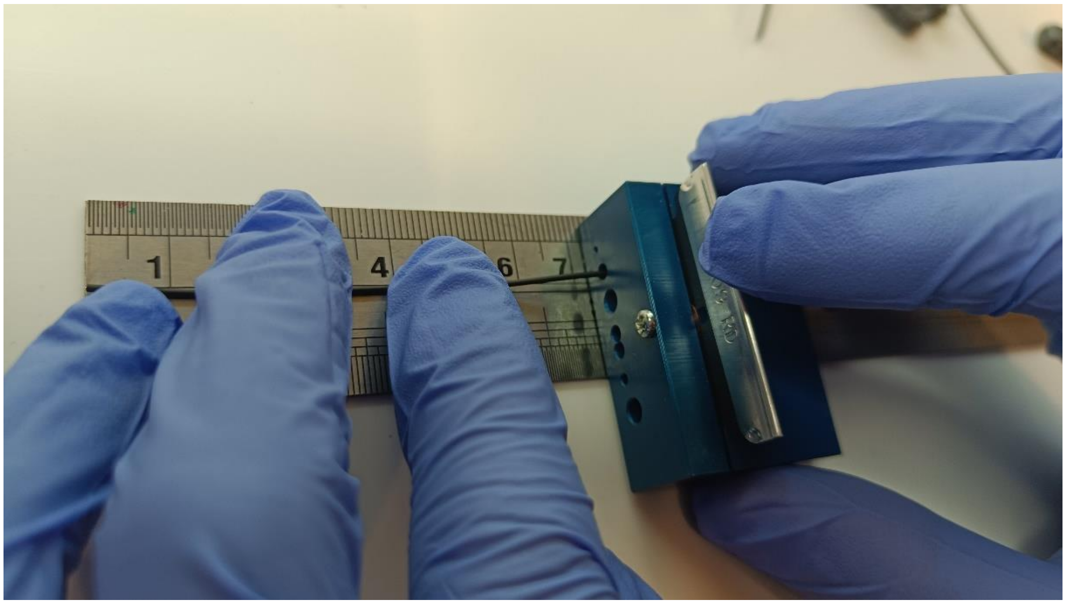

5. Guide the fibers along the 3D grooved sections of the fiber holder parts (3D parts 2 and 3).

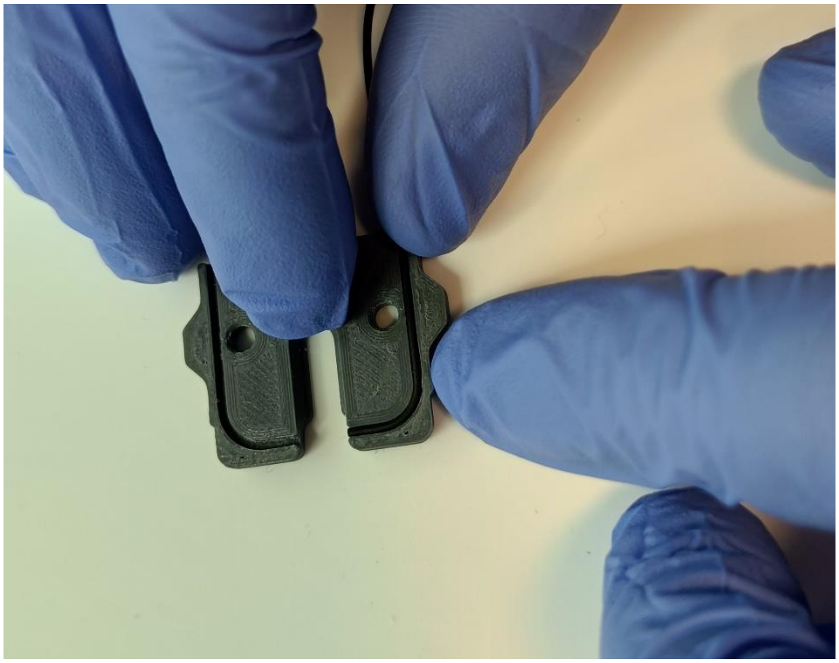

6. Introduce in the respective slots, 11x M3 square nuts. 5 placed in the 3D printed part 1 and 6 in the 3D part 4.

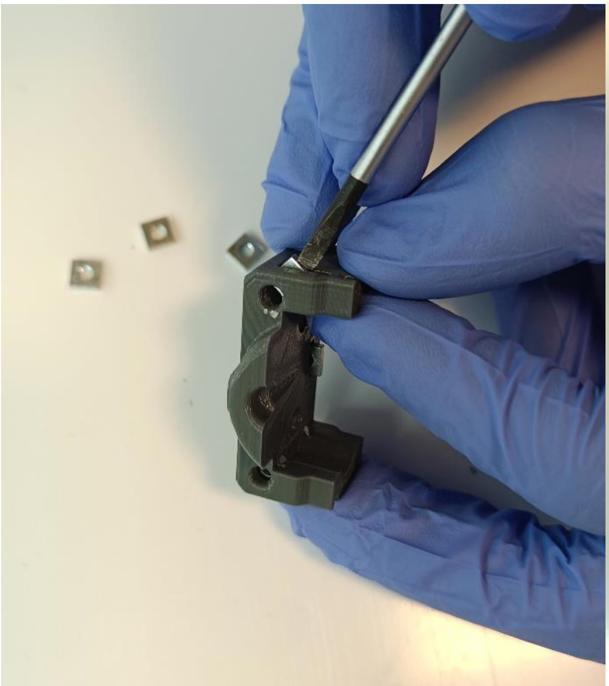

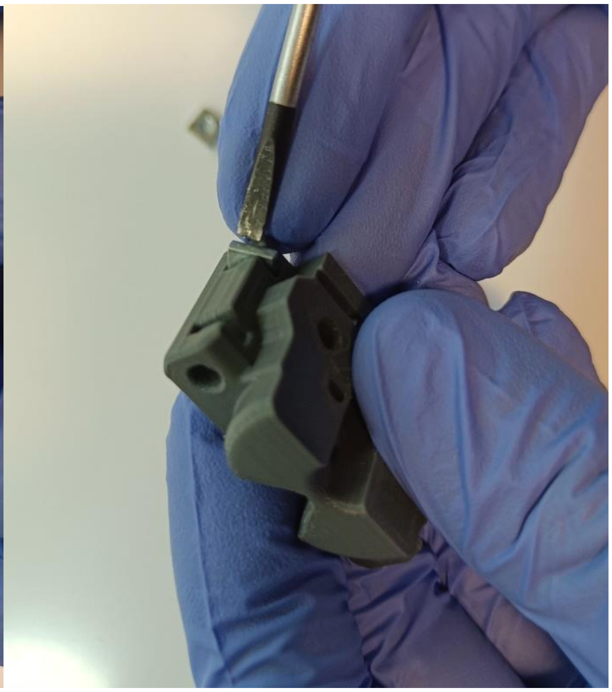

7. Assemble together the 3D parts 1,2,3 and 4 and screw them using 2x M3 20 mm length hex socket screws.

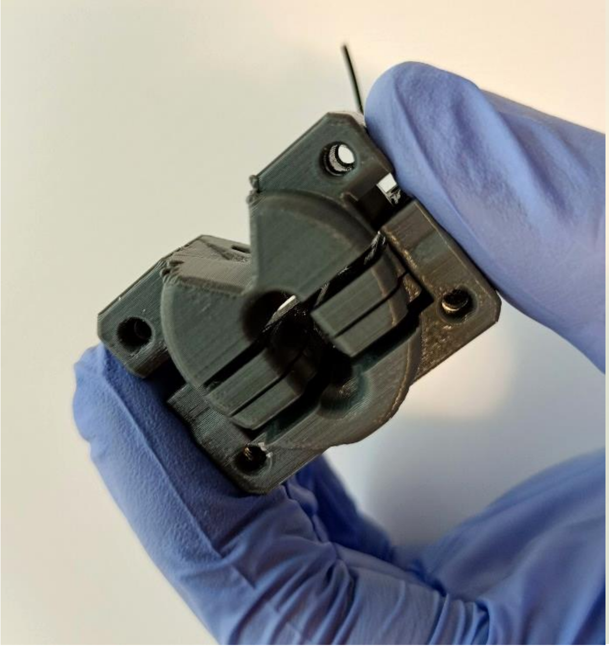 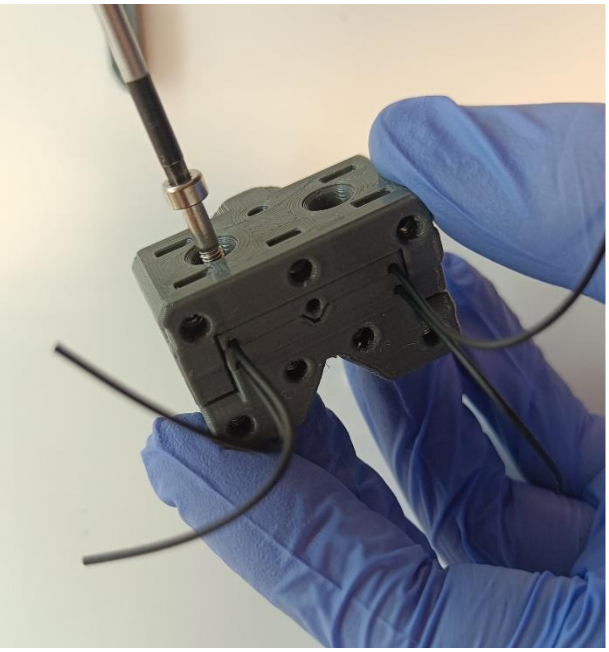

8. Attach the 3D part 5 for holding the LED int the previous assembly, using 2x M3 10 mm length nylon spacers.

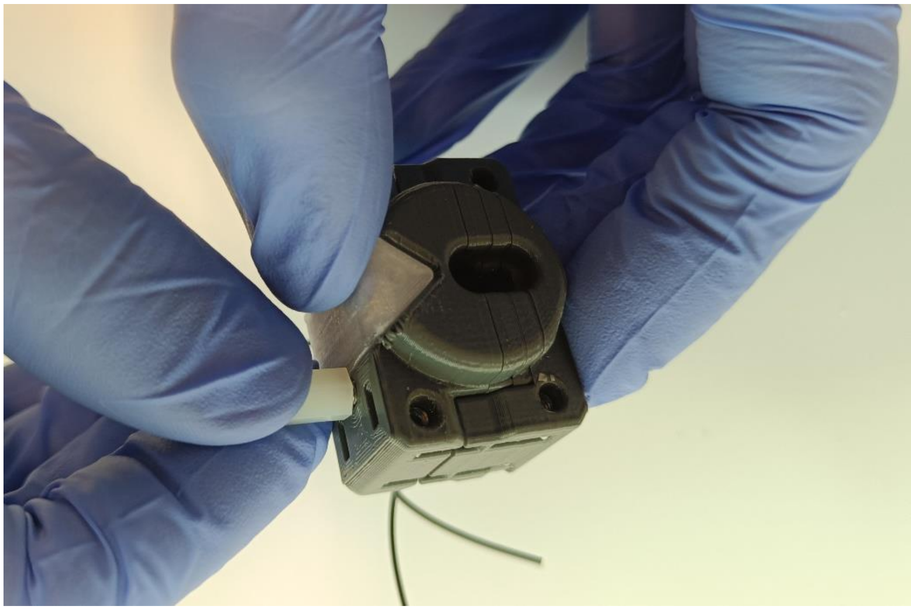

9. Attach the 3D printed assembled parts to the electronic board, using 4x M3 12 mm nylon screws.

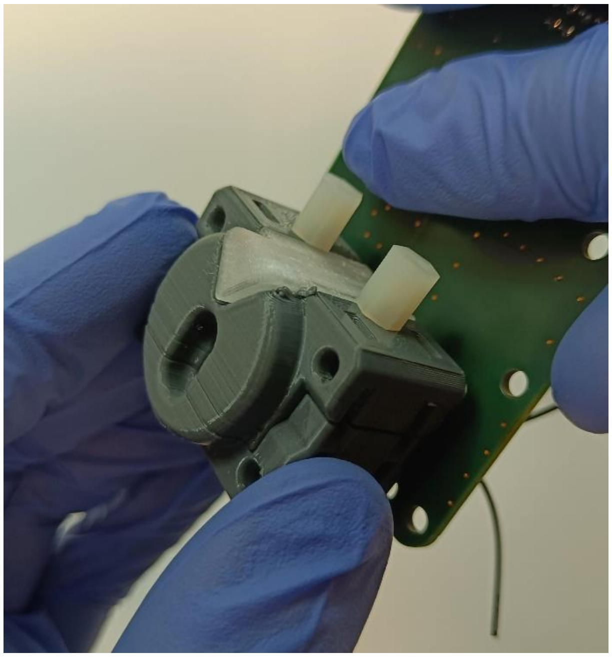

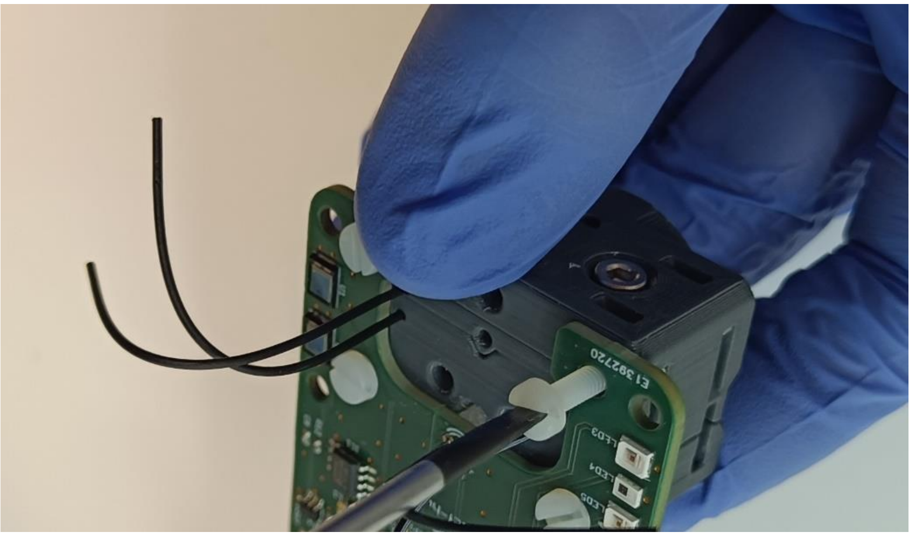

10. Introduce the end of the optical fibers into the respective holes of the 3D parts 6 and 7. If necessary use a drill to remove any imperfections from the 3D printing. The fiber should fit tight into the holes and face the other end of the 3D parts, so that is positioned as closed as possible to LED emitters and photo receivers.

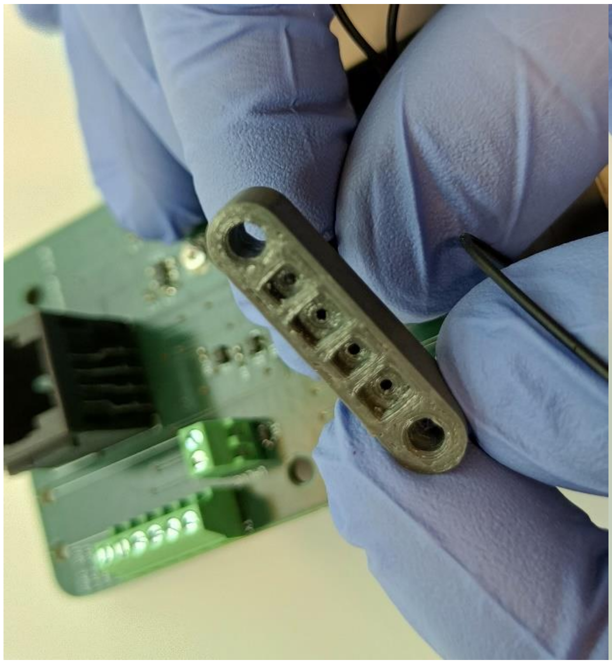
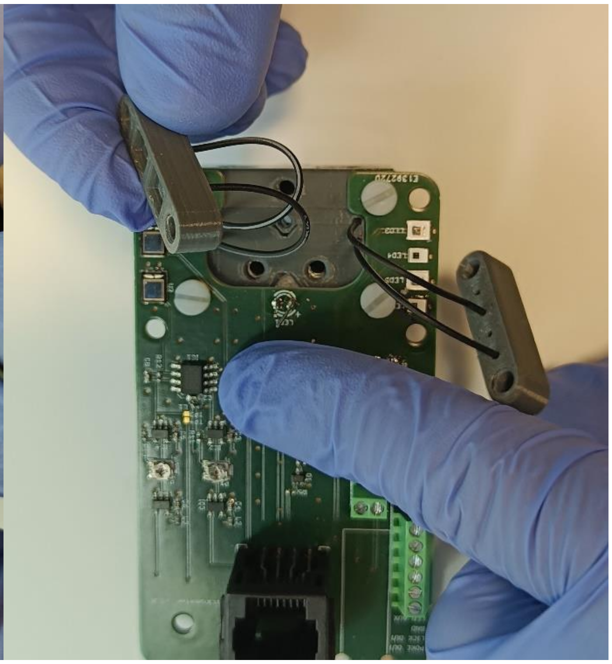

11. Screw the 3D printed parts 6 and 7 into the electrical board using 4x M3 12 mm nylon screws and 4x M3 nylon nuts.

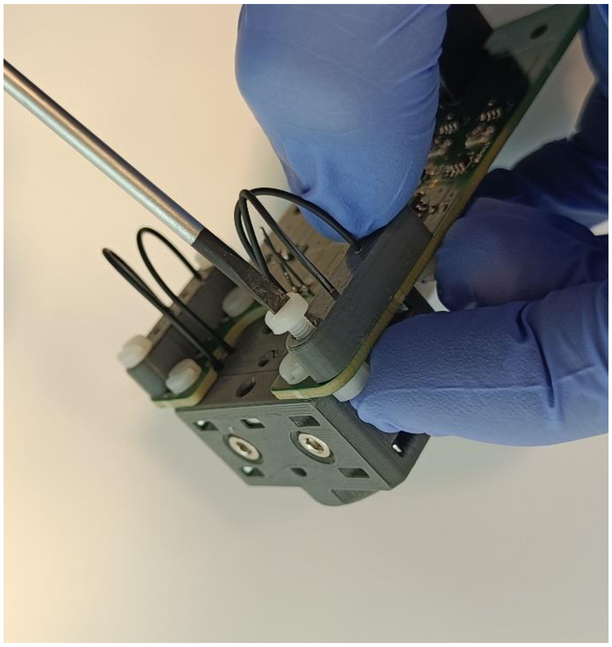

12. Attach the acrylic parts to the poke assembly using 4x M3 12 mm length countersunk screws to attach the acrylic parts.

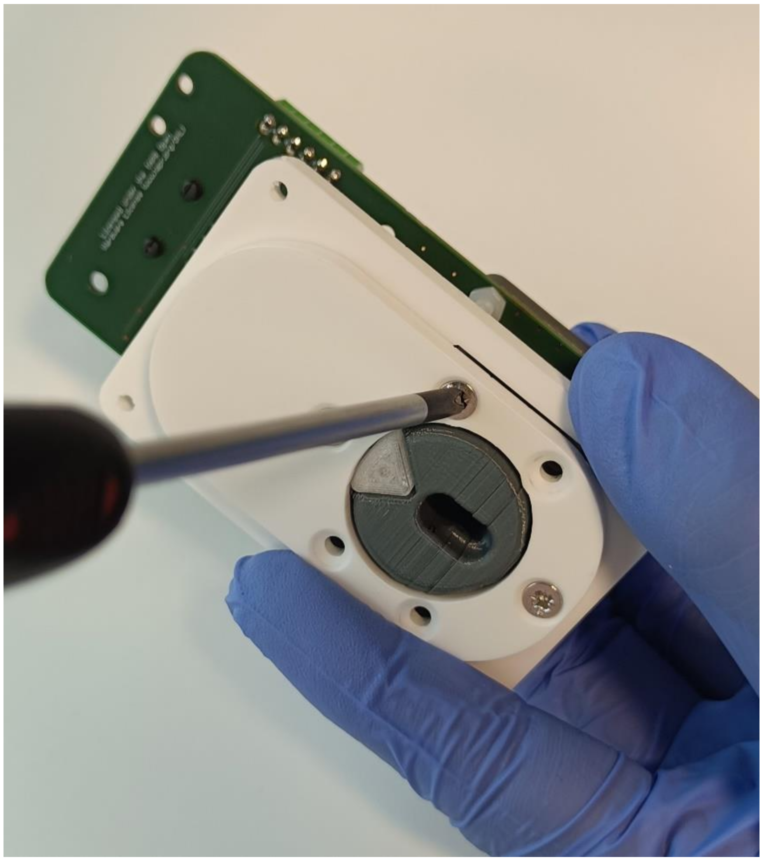

13. Attach a 16G blunt-end tip needle (847.356.0321, SAI Infusion Technologies) through the back of the assembled part. The users have the option to trim the spout and attach directly to the water delivery tube or utilize it as it is. through a Luer lock fitting.
    Optionally the 3D printed part 8 can also be attached using 3x M3 12 mm length countersunk screws – this works as a spout friction controller in the case where the spout is too loose. A 1.6 mm inside diameter silicone tubing or similar size O-Ring, with a small section is placed between the 3D poke and the 3D part 8, constraining the spout. The friction can be adjusted through the countersunk screws that are attached to the friction controller part.

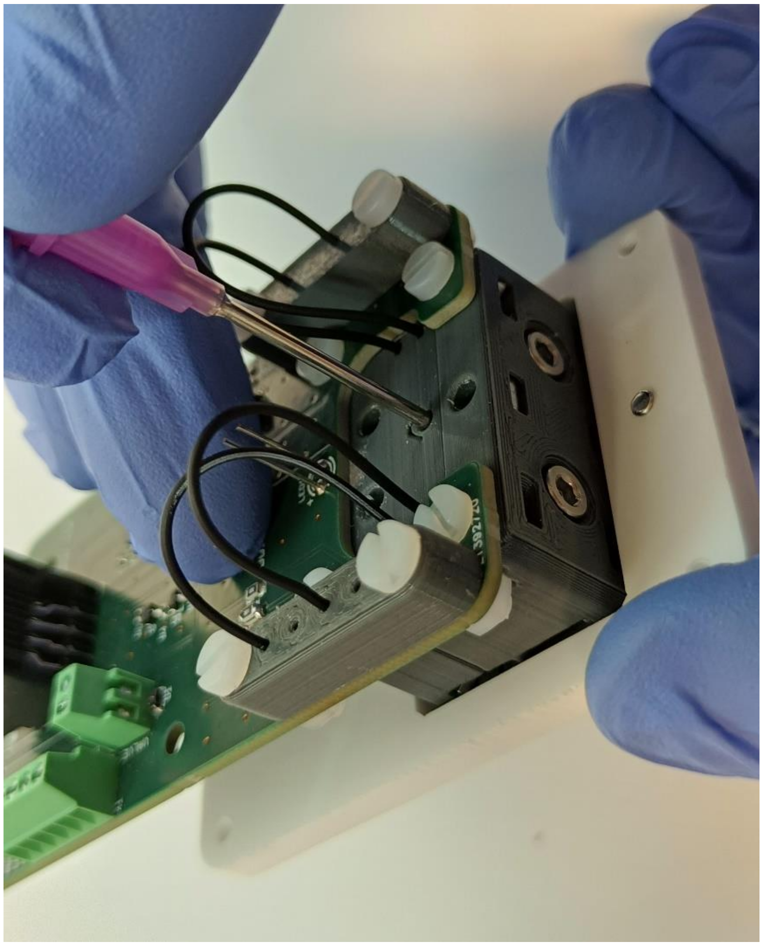

14. Following the assembly, the alignment and coupling efficiency, due to the positioning of the fibers, can be validated by measuring the voltage at the output of the transimpedance amplifiers when the beam is not broken. Measure TP3 and TP1 on the PCB. It is recommended to have a value higher than 3 V, otherwise the fibers should be repositioned.

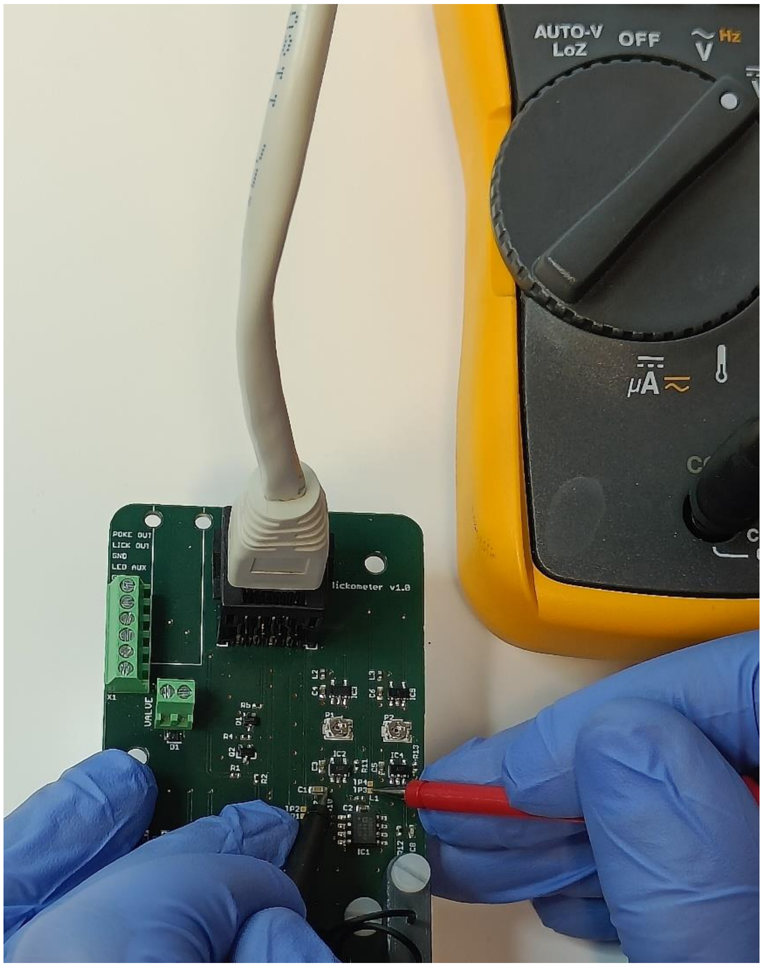
AUTO-V
LoZ
OFF
Hz
POKE OUT
LICK OUT
GND
LED AUX
lickometer v1.0
VALVE

Assembled Optical Lickometer

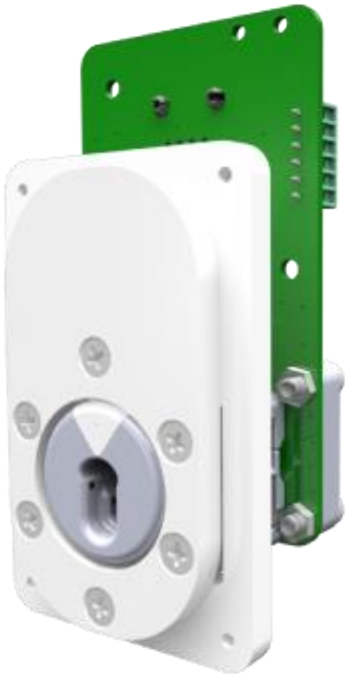

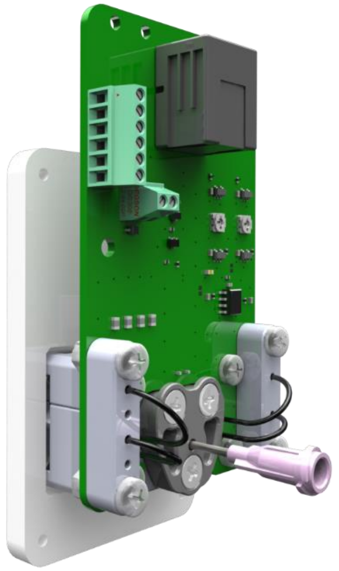

Supplement: Extended Data — Design files and assembly instructions for the mechanical parts and printed circuit board of the proposed optical lickometer. Download Extended Data, ZIP file. [file eneuro-11-ENEURO.0189-24.2024-s004.zip › Hardware/CAD/Assembly instructions.pdf]
