# Supplementary material for: High-Precision Optical Fiber-Based Lickometer
Source: eNeuro. 2024 Jul 16;11(7):ENEURO.0189-24.2024. doi: 10.1523/ENEURO.0189-24.2024 (PMC11258538; doi:10.1523/ENEURO.0189-24.2024)
Supplement: Extended Data — Design files and assembly instructions for the mechanical parts and printed circuit board of the proposed optical lickometer. Download Extended Data, ZIP file. [file eneuro-11-ENEURO.0189-24.2024-s004.zip › Hardware/PCB/lickometer v1.0 - brd.pdf]

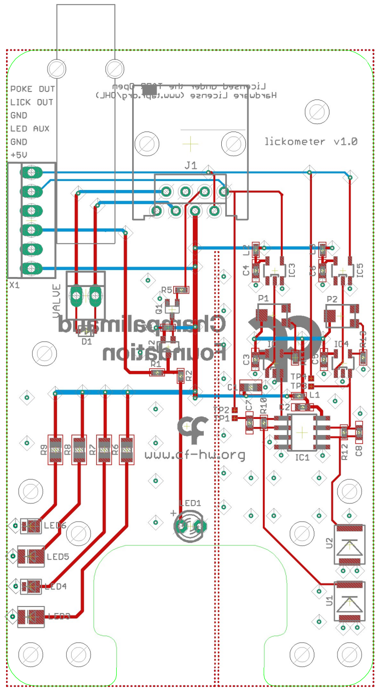

Licensed under the TAPR Open
Hardware License (www.tapr.org/OHL)

Copyright 2022 Artur Silva
Champalimaud Foundation
